# Understanding the relationship between type-2 diabetes, MRI markers of neurodegeneration and small vessel disease, and dementia risk: a mediation analysis

**DOI:** 10.1007/s10654-023-01080-7

**Published:** 2024-01-08

**Authors:** Leslie Grasset, Eric Frison, Catherine Helmer, Gwénaëlle Catheline, Geneviève Chêne, Carole Dufouil

**Affiliations:** 1 University of Bordeaux, INSERM, Bordeaux Population Health Research Center, UMR 1219, CIC1401-EC, F-33000 Bordeaux, France; 2 Service d’Information Médicale, CHU Bordeaux, Bordeaux, France; 3 INCIA, EPHE, CNRS, Université PSL, University of Bordeaux, 33076 Bordeaux, France; 4 Pole de sante publique Centre Hospitalier Universitaire (CHU) de Bordeaux, 33000 Bordeaux, France; 5 INSERM U1219, University of Bordeaux, 146 rue Léo Saignat, 33077 Bordeaux cedex, France

**Keywords:** Type-2 diabetes, Dementia, Brain MRI markers, Mediation

## Abstract

To explore to which extent neurodegeneration and cerebral small vessel disease (SVD) could mediate the association between type-2 diabetes and higher dementia risk. The analytical sample consisted in 2228 participants, out of the Three-City study, aged 65 and older, free of dementia at baseline who underwent brain MRI. Diabetes was defined by medication intake or fasting or non-fasting elevated glucose levels. Dementia status was assessed every 2 to 3 years, during up to 12 years of follow-up. Brain parenchymal fraction (BPF) and white matter hyperintensities volume (WMHV) were selected as markers of neurodegeneration and cerebral SVD respectively. We performed a mediation analysis of the effect of baseline BPF and WMHV (mediators) on the association between diabetes and dementia risk using linear and Cox models adjusted for age, sex, education level, hypertension, hypercholesterolemia, BMI, smoking and alcohol drinking status, APOE-ε4 status, and study site. At baseline, 8.8% of the participants had diabetes. Diabetes (yes vs. no) was associated with higher WMHV (β_diab_ = 0.193, 95% CI 0.040; 0.346) and lower BPF (β_diab _= −0.342, 95% CI −0.474; −0.210), as well as with an increased risk of dementia over 12 years of follow-up (HR_diab _= 1.65, 95% CI 1.04; 2.60). The association between diabetes status and dementia risk was statistically mediated by higher WMHV (HRdiab=1.05, 95% CI 1.01; 1.11, mediated part = 10.8%) and lower BPF (HR_diab _= 1.12, 95% CI 1.05; 1.20, mediated part = 22.9%). This study showed that both neurodegeneration and cerebral SVD statistically explained almost 30% of the association between diabetes and dementia.

**Supplementary Information:**

The online version contains supplementary material available at 10.1007/s10654-023-01080-7.

## Introduction

Type-2 diabetes is a frequent chronic disease, estimated to affect around 537 million adults aged 20 to 79 years old worldwide [1]. It is a known risk factor for cognitive decline, dementia, and Alzheimer’s disease (AD) at older ages [2–4]. Its population attributable fraction for dementia was estimated at 1.1% from the Lancet commission after summarizing the existing evidence [5]. Type-2 diabetes (noted as diabetes in the following) can be prevented; however, clinical trials so far were not conclusive to demonstrate an effect of diabetes control or treatment on the course of cognitive decline or dementia risk [6, 7].

The mechanisms underlying the diabetes-dementia association are various but still unclear and require further investigation. Diabetes is characterized by the presence of chronic hyperglycaemia resulting from insulin dysregulation, which also leads to inflammation or oxidative stress [8]. These physiopathological mechanisms have been implicated in cerebral small vessel disease and neurodegeneration [9]. Indeed, associations of diabetes with higher white matter hyperintensities volume, brain infarcts, lower brain volumes or cerebral atrophy, have been reported from either cross-sectional or longitudinal studies [10–17]. Cerebral small vessel disease and neurodegeneration are in turn involved in the progression of cognitive impairment and dementia. Yet, few studies have investigated markers of brain pathologies that could mediate the effect of diabetes on cognitive functions [18, 19], cognitive decline [20, 21], or dementia risk [22], and the specific roles of neurodegeneration and cerebral small vessel disease in these physiopathological pathways remain unclear. Only two studies have looked at markers of neurodegeneration and cerebral small vessel disease together: a cross sectional report from 4206 older adults (mean age 76) from an Icelandic population-based study showed that both small vessel disease and neurodegeneration markers mediated the association between diabetes and cognitive function [18], while a clinical study of 2288 patients (mean age 71) with subjective cognitive complaints (SCC) or mild cognitive impairment (MCI) reported a mediated association through neurodegeneration only [19]. Disentangling the contribution of each of these brain pathological processes is thus needed to better understand the physiopathology underlying the diabetes-dementia relationship.

The main objective of this work was to explore, in a large population-based cohort of participants free of dementia at entry and followed up over 12 years, whether the association of diabetes with dementia risk is mediated by brain MRI markers of neurodegeneration or cerebral small vessel disease.

## Research design and methods

### Study population

The design and methods for this study were reported previously [23]. The 3 C MRI substudy (Three-City MRI) is a population-based cohort study of noninstitutionalized individuals aged 65 to 80 years who were recruited in the French cities of Dijon and Bordeaux. Enrollment for the baseline MRI assessment occurred from 1999 to 2001 in 2554 persons that were thereafter followed every 2 or 3 years over a 12-year period. Repeated cognitive evaluations and active diagnosis of dementia cases have been realized. At each study wave, a standardized questionnaire assessing socio-demographic, medical, cognitive, and functional characteristics was administered at home by trained neuropsychologists during face-to-face interviews. Blood samples were also collected for assessment of standard biology.

From 2554 dementia-free participants who had baseline MRIs, 326 were excluded from analyses for missing measures of white matter hyperintensities volume or brain volumes (n = 153), missing diabetes status (n = 46), missing adjustment covariates (n = 63), or absence of follow-up (n = 102), leaving a final sample size of 2228 participants.

### Diabetes definition

Diabetes cases at baseline were defined as either 1/ Presence of fasting blood glucose ≥ 7 mmol/L (≥ 126 mg/dL) or non-fasting blood glucose ≥ 11.1 mmol/L (≥ 200 mg/dL); or 2/ Antidiabetic drug intake (Anatomical Therapeutic Chemical classification system: code A10A “insulins and analogues”, and code A10B “blood glucose lowering drugs, excl. insulins”).

### Dementia assessment

Dementia diagnosis was realized using a standardized three-step procedure. The first step was a cognitive evaluation by trained neuropsychologists using a series of psychometric tests. Participants who were suspected of dementia, based on their neuropsychological performance or decline relative to a previous examination were then examined for further medical assessments. Finally, each case was discussed by a validation committee composed of neurologists and geriatricians to classify etiology. The diagnosis of dementia was based on the Diagnostic and Statistical Manual of Mental Disorders-Fourth Edition (DSM-IV) criteria. Dementia subtyping was based on the National Institute of Neurological and Communicative Disorders and Stroke–Alzheimer’s Disease and Related Disorders Association (NINDS–ADRDA) criteria for AD, and on the National Institute of Neurological Disorders and Stroke–Association Internationale pour la Recherche et l’Enseignement en Neurosciences (NINDS–AIREN) criteria for vascular dementia. Mixed dementia was defined as a diagnosis of AD with either cerebrovascular lesions on brain imaging when available or a documented history of stroke and the presence of prominent executive function deficits in addition to an AD-type cognitive profile. We considered all incident cases that occurred during the 12-year follow-up period for the current analyses.

### Brain MRI markers

The protocol for cranial MRI, using either a 1.5-T Magnetom (Siemens, Erlangen, Germany) in Dijon or an ACHIEVA 3T scanner (Philips Medical System, Netherlands) in Bordeaux, has been described in detail previously [24]. For this study, we selected the two following MRI markers, available in both study sites: brain parenchymal fraction (BPF) as a marker of global atrophy, and white matter hyperintensities volume (WMHV) as a marker of small vessel disease burden. Using voxel-based morphometry techniques, BPF was calculated as the sum of grey and white matter volumes minus white matter hyperintensities volume divided by total intracranial volume (sum of grey and white matter volumes and cerebrospinal fluid volume). A fully automatic image processing software was developed for tissue segmentation and to detect and quantify white matter hyperintensities [24]. WMHV was calculated by summing the volumes of all the lesions detected. WMHV was divided by total white matter volume and log transformed. In the following analyses, BPF and WMHV were standardized for both sites separately.

### Covariates

At baseline, sociodemographic information was collected that included age, sex, and education level (categorized as no or primary school, secondary school, high school, university). Potential confounding factors to be controlled for included: study site (Bordeaux vs. Dijon), smoking status (never, former, and current smoker), alcohol consumption (never, former, and current drinker), self-reported history of cardiovascular disease (myocardial ischemia, coronary artery disease or peripheral arterial disease) and stroke, hypertension, hypercholesterolemia, body mass index (BMI), and depressive symptoms. Hypertension was defined by either measured systolic blood pressure ≥ 140 mmHg or diastolic blood pressure ≥ 90 mmHg, antihypertensive drug intake, or self-reported history of hypertension. Hypercholesterolemia was defined by either total cholesterol level ≥ 7.25 mmol/L, lipid-lowering drug intake, or self-reported history of hypercholesterolemia. BMI was categorized as < 20 kg/m² (underweight), 20 to 24.9 kg/m² (normal weight), 25 to 29.9 kg/m² (overweight) and ≥ 30 kg/m² (obesity). Depressive symptomatology was assessed with the Center for Epidemiological Studies-Depression (CESD) scale, using scores of > 16 for men and > 22 for women as indicators of a clinically relevant level of depressive symptomatology [25]. Finally, APOE-ε4 status was defined as at least one ε4 allele carried versus none [26]. These covariates were selected because we hypothesized they may be confounders of the relationship between diabetes and dementia (Exposure–Outcome), diabetes and WMHV (Exposure–Mediator1), diabetes and BPF (Exposure–Mediator2), and/or WMHV/BPF and cognition (Mediator1/2–Outcome).

### Statistical analysis

Comparisons of participants included in the analytical sample versus excluded were realized for baseline characteristics. In the analytical sample, participants’ characteristics at baseline according to diabetes status were reported using median and interquartile range, as well as numbers and proportions. Comparisons were realized using Wilcoxon tests for continuous variables, and χ^2^ for categorical variables.

**Fig. 1:**
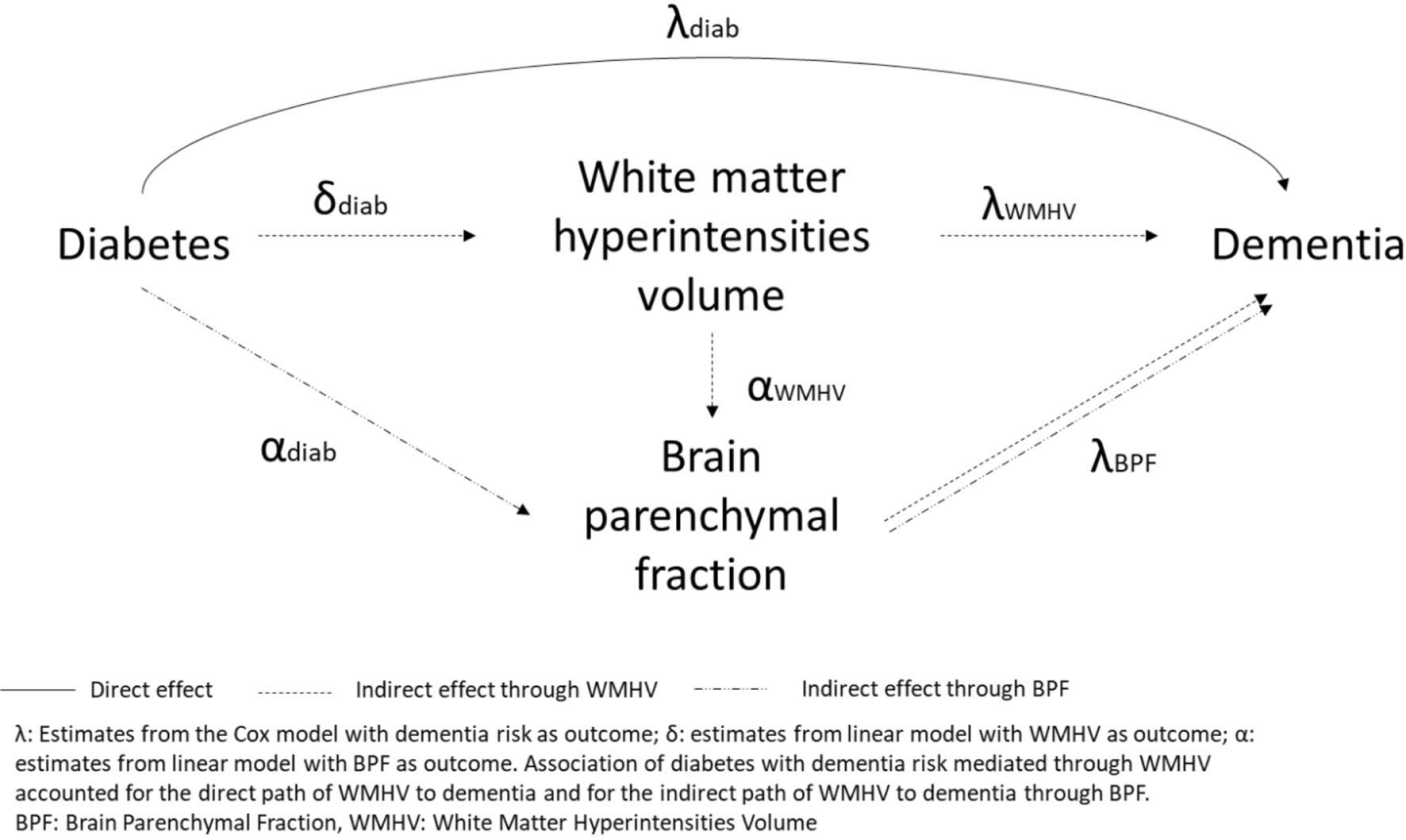
Mediation model of the association between diabetes and dementia risk

We performed a mediation analysis with diabetes status at baseline as main exposure, baseline BPF and WMHV as mediators, and dementia onset (as well as AD dementia onset comprising AD dementia cases and mixed dementia cases) as the outcome (Fig. 1). In our conceptual model, a causal relationship between WMHV and BPF was hypothesized [27–31]. The pathway mediated by WMHV thus accounted for the direct effect of WMHV on dementia as well as its indirect effect through BPF. Causal mediation analyses require four important assumptions on confounders: (a) no unmeasured exposure–outcome confounding, (b) no unmeasured exposure–mediator confounding, (c) no unmeasured mediator–outcome confounding, and (d) no mediator–outcome confounder is affected by the exposure [32]. After inclusion of major confounders, we considered these assumptions as reasonable. For this causal mediation analysis of survival outcome with multiple mediators, we used the parametric method proposed by Huang et al. [33] to estimate total, direct, and path-specific indirect effects between diabetes and dementia risk, and their associated 95% confidence intervals. In a first step, cross sectional associations of diabetes with BPF and WMHV were estimated using two multivariable linear regression models adjusted for age, sex, education level, hypertension, hypercholesterolemia, BMI, smoking and alcohol drinking status, APOE-ε4 status, and study site. The model with BPF as an outcome was additionally adjusted for WMHV. In a second step, delayed entry Cox proportional hazard models, with age as time scale, were used to estimate the association between diabetes and dementia risk. Participants who remained free of dementia over the follow-up length were censored at the age of their last follow-up before drop-out or at the end of follow up. Models were adjusted for sex, education level, hypertension, hypercholesterolemia, BMI, smoking and alcohol intake status, APOE-ε4 status, and study site. Then, the Cox model for dementia risk was additionally adjusted for both brain MRI markers. We tested a potential exposure–mediators interaction, which was not significant. Total effect consisted in the sum of the direct and each path-specific indirect effect. The proportion of mediation by each brain marker was calculated as the ratio of each indirect effect to the total effect. Risk proportionality was assessed graphically and with Schoenfeld’s residuals testing. Confidence intervals were calculated using a resampling method that takes random draws (n = 10^6^) from multivariate normal distribution of the different estimates and their covariance matrix (see Appendix sections A4 and A5 of Huang et al. for reference [33]). Empirical 95% confidence intervals corresponded to the 2.5 and 97.5 percentiles of the obtained distributions.

Sensitivity analyses were realized to evaluate the robustness of our results. First, because diabetes is associated with higher risk of death, competing risk of death may influence the results. We thus performed an illness-death model instead of the initial Cox model. Such model accounts for competing risk of death as well as interval censoring. Second, the primary results reported were based on a complete cases analysis under the assumption that missing data at baseline that led to exclude participants from the final analyses (n = 224) were missing completely at random (MCAR). To further explore the impact of missing data being MCAR on our findings, missing values of diabetes, MRI markers, and covariates were imputed by multiple imputation (MI) using chained equations with a fully conditional specification (10 imputed data sets), and the primary analysis was rerun on those imputed datasets. Third, due to baseline imbalance of depressive symptoms between participants with and without diabetes, we also performed the primary analysis adjusting for high depressive symptoms. Fourth, to investigate whether the link between WMHV and BPF influenced our results, we performed an analysis without accounting for this association. Then, to account for potential residual confounding due to an unmeasured common cause for the mediators, we estimated the joint indirect effect through both mediators using the Inverse Odds Weighting method [34, 35] composed of 3 steps. First, a logistic regression modelling the probability of diabetes, adjusted for mediators and confounding factors, was used to create a weight equal to (1-predicted probability)/predicted probability for diabetics and to 1 for non-diabetics. Second, we estimated diabetes direct effect with a weighted Cox model, adjusted for confounding factors only. Third, total effect was estimated using an unweighted Cox model, adjusted for confounding factors only. 95% confidence intervals for each total, direct, and indirect effects were calculated using bootstrap (1000 replications). The proportion of mediated effect was then compared with the sum of the path-specific effects from the primary analysis.

Finally, to further assess the potential impact of residual confounding, we provided E values and the associated lower limit of the 95% confidence interval (LCI), for the association between diabetes and dementia. Briefly, an E value expresses the minimum strength of association that an unmeasured confounder needs to reach with both the exposure and the outcome to fully explain away a specific exposure–outcome association. E values and associated LCI were calculated using the E value calculator [36, 37]. In addition, regarding robustness to potential unmeasured mediators–outcome confounding, we used a mediational E value as developed by Smith and VanderWeele [38].

Analyses were conducted using SAS v9.4 (SAS Institute Inc.), and R version 4.1.3 [39].

## Results

Compared to participants included in the analytic sample, participants excluded were on average older, and had on average lower MMSE scores. They also had more often comorbidities such as high BMI, hypertension, diabetes or high depressive symptoms. (Online Resource 1) Characteristics of analytical sample (n = 2228) by diabetes status (present in n = 181, 8.1% of participants) are presented in Table 1. Participants with diabetes at baseline were more often male and had more frequently other cardiovascular comorbidities as well as high depressive symptoms.

**Table 1:** Population’s characteristics description according to diabetes status, the 3 C study (Bordeaux + Dijon)

	No diabetes (n = 2047)	Diabetes (n = 181)	*P* value
Dijon study site	1535 (75.0)	139 (76.8)	0.59
Age at baseline	72.1 [68.8–75.8]	72.2 [68.9–75.7]	0.50
Female	1257 (61.4)	83 (45.9)	< 0.001
Education level			0.32
No diploma or primary school	625 (30.5)	61 (33.7)	
Secondary school	620 (30.3)	61 (33.7)	
High school	247 (12.1)	21 (11.6)	
University	555 (27.1)	38 (21.0)	
APOE-ε4 carriers	442 (21.6)	37 (20.4)	0.72
Smoking status			< 0.001
Never smoker	1286 (62.8)	82 (45.3)	
Former smoker	653 (31.9)	89 (49.2)	
Current smoker	108 (5.3)	10 (5.5)	
Drinking status			0.47
Non drinker	343 (16.8)	30 (16.6)	
Former drinker	40 (1.9)	6 (3.3)	
Drinker	1664 (81.3)	145 (80.1)	
Baseline MMSE score	28.0 [27.0–29.0]	28.0 [26.0–29.0]	0.01
BMI			< 0.001
< 20	110 (5.4)	4 (2.2)	
20–24.9	876 (42.8)	45 (24.9)	
25–29.9	828 (40.4)	87 (48.1)	
≥ 30	233 (11.4)	45 (24.9)	
History of CVD	129 (6.3)	24 (13.3)	< 0.001
History of stroke	77 (3.8)	9 (5.0)	0.53
Hypertension	1531 (74.8)	160 (88.4)	< 0.001
Hypercholesterolemia	883 (43.1)	104 (57.5)	< 0.001
High depressive symptoms	207 (10.1)	30 (16.1)	0.03
Glycaemia	4.84 [4.54–5.18]	7.0 [5.4–8.3]	< 0.001

Data presented being N (frequency) or median [IQR]

MMSE, Mini Mental State Examination; APOE, apolipoprotein E; CVD, Cardiovascular disease; BMI, Body Mass Index

*p-value: Wilcoxon for quantitative variable or χ2 for qualitative variables

Diabetes was associated with higher WMHV (β = 0.210, 95% CI 0.057; 0.363) and lower BPF (β = − 0.323, 95% CI − 0.457; − 0.189). In addition, higher WMHV was associated with lower BPF (β = − 0.137, 95% CI − 0.174; − 0.101). Over a median follow-up of 8.6 years (IQR 5.1–10.7), 196 participants were diagnosed with dementia (incidence rate: 1.09/100 persons-years (95% CI 0.93; 1.24). In a covariates-adjusted delayed entry Cox model, diabetes at baseline was associated with an increased risk of dementia over 12 years of follow-up (HR = 1.65, 95% CI 1.04; 2.60) (Table 2). After additional adjustment for MRI markers, this association was attenuated and became statistically non-significant (HR = 1.42, 95% CI 0.89; 2.25). Higher WMHV was associated with higher risk of dementia (HR = 1.26, 95% CI 1.10; 1.41), while higher BPF was associated with lower risk of dementia (HR = 0.78, 95% CI 0.66; 0.91) (Table 2). Results from the sensitivity analysis accounting for competing risk of death showed slightly lower estimates for the associations between diabetes and dementia, and similar estimates for the associations between mediators and dementia. Diabetes was associated with a 46% increased risk of AD dementia but did not reach statistical significance (HR = 1.46, 95% CI 0.86 ; 2.49).

**Table 2:** Associations between diabetes status and dementia risk, the 3 C study (Bordeaux + Dijon)

	Adjusted model *		Model additionally adjusted for mediators	
	HR (95% CI)	*P* value	HR (95% CI)	*P* value
**Dementia**				
Diabetes	1.65 (1.04–2.60)	0.03	1.42 (0.89–2.25)	0.14
WMHV (+ 1 SD)	–		1.26 (1.10–1.44)	< 0.001
BPF (+ 1 SD)	–		0.78 (0.66–0.91)	0.002
**AD dementia**				
Diabetes	1.46 (0.86–2.49)	0.17	1.29 (0.79–2.20)	0.36
WMHV (+ 1 SD)	–		1.19 (1.02–1.39)	0.02
BPF (+ 1 SD)	–		0.80 (0.67–0.96)	0.01
**Dementia accounting for competing risk of death**^a^				
Diabetes	1.61 (1.02–2.54)	0.04	1.38 (0.87–2.18)	0.17
WMHV (+ 1 SD)	–		1.25 (1.09–1.43)	0.001
BPF (+ 1 SD)	–		0.75 (0.64–0.88)	< 0.001

Hazard ratios correspond to the associations between diabetes and each mediator with dementia risk

WMHV, White matter hyperintensities volume; BPF, Brain parenchymal fraction

*Model adjusted for sex, education level, smoking and drinking status, BMI, hypertension, hypercholesterolemia, APOE-ε4 status, and study site

^a^ Using illness-death models

The association between diabetes status and dementia risk was significantly mediated by higher WMHV (HR_indirect_WMHV_=1.06, 95% CI 1.01; 1.12, mediated part = 11.4%) and lower BPF (HR_indirect_BPF_=1.09, 95% CI 1.03; 1.26, mediated part = 16.9%) (Table 3). The total proportion of mediated effect by both brain MRI markers was 28.3%. Results from sensitivity analyses are presented in Table 3. The analysis using imputed data yielded stronger total and direct effect and similar indirect effect, thus lowering the mediated proportion to 22.8%. Analyses further controlling for high depressive symptoms and not accounting for the link between WMHV and BPF yielded similar results (mediated proportions = 26.4% and 28.3% respectively). When using the Inverse Odds Weighting approach, the joint mediated proportion was 37.5%, higher compared to the primary analysis.

**Table 3:** Mediated associations between diabetes status and dementia risk, the 3 C study (Bordeaux + Dijon)

	HR (95% CI)	Mediated proportion
**Dementia**		
Total effect	1.63 (1.02–2.58)	
Direct effect	1.42 (0.89–2.25)	71.7%
Indirect effect		
By BPF	1.09 (1.03–1.16)	16.9%
By WMHV	1.06 (1.01–1.12)	11.4%
**Sensitivity analysis 1**		
Total effect	1.81 (1.24–2.66)	
Direct effect	1.58 (1.08–2.32)	77.2%
Indirect effect		
By BPF	1.09 (1.03–1.15)	14.0%
By WMHV	1.05 (1.01–1.10)	8.8%
**Sensitivity analysis 2**		
Total effect	1.64 (1.03–2.61)	
Direct effect	1.44 (0.91–2.29)	73.6%
Indirect effect		
By BPF	1.08 (1.02–1.16)	15.9%
By WMHV	1.05 (1.01–1.11)	10.5%
**Sensitivity analysis 3**		
Total effect	1.63 (1.03–2.58)	
Direct effect	1.42 (0.89–2.25)	71.7%
Indirect effect		
By BPF	1.09 (1.03–1.18)	18.4%
By WMHV	1.05 (1.01–1.11)	9.9%
**Sensitivity analysis 4**		
Total effect	1.65 (0.95–2.59)	
Direct effect	1.37 (0.67–2.80)	
Indirect effect	1.21 (0.69–1.92)	37.5%

WMHV, White matter hyperintensities volume; BPF, Brain parenchymal fraction

Models adjusted for sex, education level, smoking and drinking status, BMI, hypertension, hypercholesterolemia, APOE-ε4 status, and study site. Sensitivity analysis 1: similar adjustment as main analysis with multiple imputation for missing data. Sensitivity analysis 2: with adjustment for high depressive symptoms. Sensitivity analysis 3: without accounting for the association between WMHV and BPF. Sensitivity analysis 4: using Inverse Odds Weighting method.

Hazard ratios correspond to total and direct effect of diabetes on dementia risk, as well as indirect effects of diabetes on dementia risk through BPF or WMHV.

E value calculations yielded to the conclusion that to fully explain the association between diabetes and dementia, an unmeasured confounder should be associated with both diabetes and dementia with a magnitude of at least 2.71 (LCI = 1.28). Moreover, to completely explain away the observed indirect effect, an unmeasured confounder associated with both WMHV (or BPF) and dementia risk with approximate hazard ratios of 1.31-fold each (LCI = 1.11) (or 1.40-fold (LCI = 1.21) for BPF), above and beyond the measured covariates, could suffice, but weaker confounding could not.

## Conclusions

In this prospective population-based study, we performed a mediation analysis to decipher neurodegeneration and cerebral small vessel disease mechanisms that could link diabetes to higher dementia risk. We evidenced that almost 30% of the association of diabetes with dementia risk was mediated by lower BPF and higher WMHV. These results remained consistent across the different sensitivity analyses. This work suggests that both neurodegeneration and small vessel disease, to a lower extent for the latest, play a role in the diabetes—dementia relation.

While the relationship between diabetes and cognitive impairment or dementia has been largely described [3], the underlying mechanisms remained uncertain, which justifies studies investigating pathways linking diabetes to brain and cognitive aging. Our results confirmed the association of diabetes with MRI markers of neurodegeneration, such as lower cerebral volumes or cortical atrophy [10–15, 40], and of small vessel disease [15, 40–42]. Other studies than ours also investigated the mediating role of neurodegeneration and/or cerebral small vessel disease on the association between diabetes and dementia or cognition, yet some were cross-sectional or based on clinical samples, which limited their interpretation and generalizability [14, 18–22, 42]. In a cross sectional study of 4206 older adults (mean age 76) from the Age, Gene/Environment Susceptibility–Reykjavik population-based cohort, both markers of neurodegeneration and cerebrovascular lesions mediated the association between diabetes and cognitive performances [18]. Moreover, two longitudinal studies looked at the mediating role of either neurodegenerative or cerebrovascular lesions separately: the first one using data from the Alzheimer’s Disease Neuroimaging Initiative clinical cohort showed an indirect effect of diabetes on cognitive decline through lower baseline cortical thickness, and the second one evidenced that brain hypoperfusion and white matter disease mediated the association between diabetes and either cognitive impairment or dementia in a sample derived from the population-based Cardiovascular Health Study [21, 22]. In our previous work from a different cohort of patients with SCC or MCI (the clinic-based Memento cohort), we observed in a cross-sectional analysis that the association between diabetes and lower cognitive functions was mediated by markers of neurodegeneration, but not by WMHV [19]. Similar conclusions were reported in another cross-sectional Australian study [14]. The lack of mediated association through vascular lesions in clinical settings may be due to selection in studies set up from memory clinics which tend to underrepresent persons with vascular disease who are followed up in different settings. To our knowledge, our study is the first longitudinal population-based study investigating the mediating role of both cerebral atrophy and small vessel disease markers on the association between diabetes and dementia risk. In this work, even if both white matter hyperintensities volume and brain parenchymal fraction significantly mediated the association between diabetes and dementia, we showed that the part of the association mediated through worse small vessel disease was weaker (~ 11%) than the part of the association mediated through cerebral atrophy. This could be due to the use of only one marker of cerebrovascular disease or to the fact that diabetes lead to neurodegeneration through different other unmeasured pathways. Our work thus provides additional evidence regarding the implication of different brain lesions on the impact of diabetes on pathological aging.

Taken together, these results support the hypothesis that diabetes-associated metabolic changes (impaired glucose control and insulin resistance) likely favor neurodegeneration and cerebral pathology to a lower extent. Indeed, chronic hyperglycemia is known to lead to the production of advanced glycated products, which may increase oxidative stress and inflammation [8, 43]. Diabetes, through altered insulin signaling, may also impair the permeability of the blood brain barrier [44]. These modifications may be involved with the development of macro- and micro-vascular lesions, but mostly with cerebral atrophy in dementia related areas [14, 45]. Although the direct effect of diabetes on dementia in our analysis became non-statistically significant after controlling for selected markers of neurodegeneration and cerebral small vessel disease, it was not null and other mechanisms may be involved. Secondary to insulin resistance, it has also been hypothesized that diabetes may lead to the accumulation of β-amyloid as well as tau hyperphosphorylation, both involved in increased Alzheimer’s disease risk. However, most studies failed to evidence an association between diabetes and Alzheimer’s disease related pathology, as measured by PET imaging or CSF biomarkers [19, 46]. Studies investigating other pathways independent of neurodegeneration and cerebral small vessel disease are thus required.

This study has several strengths. The results are generated from a prospective, population-based cohort with a long follow-up and large sample size. The availability of MRI markers of brain health allowed us to investigate different mediators which will improve our understanding of the relationship between diabetes and dementia. Additionally, this work relies on well-defined dementia cases, reviewed by a validation committee. This work also has some limitations. First, diabetes and mediators were measured at the same time, thus, the causal ordered relationship between diabetes, WMHV and BPF could not be verified. In this cohort, even if assessed at study entry, diabetes was diagnosed in average 11 years before baseline, limiting reverse causality. Yet, it cannot be excluded that white matter loss may lead to white matter hyperintensities (especially for periventricular white matter lesions). Since we only used one cerebral MRI marker for neurodegeneration and one for small vessel disease at one time point, the proportion mediated by each of these underlying mechanisms may have been underestimated. Selection bias is another potential limitation of our study. The sensitivity analysis using multiple imputation to account for selection yielded stronger direct effect and thus lower mediated proportion, suggesting that the characteristics specific to excluded participants (i.e. obesity, hypertension, and depressive symptoms) may also play a role in the association between diabetes and dementia through different pathways. Finally, although we accounted for multiple known confounders, we cannot exclude any remaining confusion. Yet, our conclusion held when controlling for residual confounding between the two mediators, and calculated E values suggested that our results were robust to any remaining confounding factors.

In conclusion, this work suggests that cerebral small vessel disease and neurodegeneration explained almost 30% of the effect of diabetes on dementia risk, with a smaller contribution of cerebral small vessel disease. Longitudinal studies integrating multiple dynamic brain structural and functional data will be required to confirm these results and better understand the diabetes—dementia relationship.

### Electronic supplementary material

Below is the link to the electronic supplementary material.

Supplementary Material 1

## References

[1] International Diabetes Federation. IDF Diabetes Atlas, 10th edn, Brussels. Belgium. 2021. https://www.diabetesatlas.org. Accessed June 2022.

[2] Srikanth V, Sinclair AJ, Hill-Briggs F, Moran C, Biessels GJ (2020). Type 2 diabetes and cognitive dysfunction-towards effective management of both comorbidities. Lancet Diabetes Endocrinol.

[3] Mayeda ER, Whitmer RA, Yaffe K (2015). Diabetes and cognition. Clin Geriatr Med.

[4] Dove A, Shang Y, Xu W (2021). The impact of diabetes on cognitive impairment and its progression to dementia. Alzheimers Dement.

[5] Livingston G, Huntley J, Sommerlad A (2020). Dementia prevention, intervention, and care: 2020 report of the lancet commission. Lancet.

[6] Areosa Sastre A, Vernooij RW, Gonzalez-Colaco Harmand M, Martinez G (2017). Effect of the treatment of type 2 diabetes Mellitus on the development of cognitive impairment and dementia. Cochrane Database Syst Rev.

[7] McMillan JM, Mele BS, Hogan DB, Leung AA (2018). Impact of pharmacological treatment of diabetes mellitus on dementia risk: systematic review and meta-analysis. BMJ Open Diabetes Res Care.

[8] Oguntibeju OO (2019). Type 2 diabetes mellitus, oxidative stress and inflammation: examining the links. Int J Physiol Pathophysiol Pharmacol.

[9] Biessels GJ, Despa F (2018). Cognitive decline and dementia in diabetes mellitus: mechanisms and clinical implications. Nat Rev Endocrinol.

[10] den Heijer T, Vermeer SE, van Dijk EJ (2003). Type 2 diabetes and atrophy of medial temporal lobe structures on brain MRI. Diabetologia.

[11] Debette S, Seshadri S, Beiser A (2011). Midlife vascular risk factor exposure accelerates structural brain aging and cognitive decline. Neurology.

[12] Falvey CM, Rosano C, Simonsick EM (2013). Macro- and microstructural magnetic resonance imaging indices associated with diabetes among community-dwelling older adults. Diabetes Care.

[13] Moran C, Beare R, Phan TG (2015). Type 2 diabetes mellitus and biomarkers of neurodegeneration. Neurology.

[14] Moran C, Phan TG, Chen J (2013). Brain atrophy in type 2 diabetes: regional distribution and influence on cognition. Diabetes Care.

[15] Schneider ALC, Selvin E, Sharrett AR (2017). Diabetes, prediabetes, and brain volumes and subclinical cerebrovascular disease on MRI: the atherosclerosis risk in communities neurocognitive study (ARIC-NCS). Diabetes Care.

[16] Zhang T, Shaw M, Cherbuin N (2022). Association between type 2 diabetes mellitus and brain atrophy: a meta-analysis. Diabetes Metab J.

[17] van Harten B, de Leeuw FE, Weinstein HC, Scheltens P, Biessels GJ (2006). Brain imaging in patients with diabetes: a systematic review. Diabetes Care.

[18] Qiu C, Sigurdsson S, Zhang Q (2014). Diabetes, markers of brain pathology and cognitive function: the age, gene/environment susceptibility-Reykjavik study. Ann Neurol.

[19] Frison E, Proust-Lima C, Mangin JF (2021). Diabetes Mellitus and cognition: pathway analysis in the MEMENTO cohort. Neurology.

[20] Wang R, Laveskog A, Laukka EJ (2018). MRI load of cerebral microvascular lesions and neurodegeneration, cognitive decline, and dementia. Neurology.

[21] Moran C, Beare R, Wang W, Callisaya M, Srikanth V (2019). Alzheimer’s disease neuroimaging I. Type 2 diabetes mellitus, brain atrophy, and cognitive decline. Neurology.

[22] Andrews RM, Shpitser I, Lopez O (2020). Examining the causal mediating role of brain pathology on the relationship between Diabetes and cognitive impairment: the cardiovascular health study. J R Stat Soc Ser a Stat Soc.

[23] Group CS (2003). Vascular factors and risk of Dementia: design of the three-city study and baseline characteristics of the study population. Neuroepidemiology.

[24] Maillard P, Delcroix N, Crivello F (2008). An automated procedure for the assessment of white matter hyperintensities by multispectral (T1, T2, PD) MRI and an evaluation of its between-centre reproducibility based on two large community databases. Neuroradiology.

[25] Radloff L (1977). The CES-D, scale: a self-report depression scale for research in the general population. Appl Psychol Meas.

[26] Dufouil C, Richard F, Fievet N (2005). APOE genotype, cholesterol level, lipid-lowering treatment, and dementia—the three-city study. Neurology.

[27] Rizvi B, Narkhede A, Last BS (2018). The effect of white matter hyperintensities on cognition is mediated by cortical atrophy. Neurobiol Aging.

[28] Godin O, Maillard P, Crivello F (2009). Association of white-matter lesions with brain atrophy markers: the three-city Dijon MRI study. Cerebrovasc Dis.

[29] Tosto G, Zimmerman ME, Hamilton JL, Carmichael OT, Brickman AM, Alzheimer’s Disease Neuroimaging Initiative (2015). The effect of white matter hyperintensities on neurodegeneration in mild cognitive impairment. Alzheimers Dement.

[30] Keuss SE, Coath W, Nicholas JM (2022). Associations of beta-amyloid and vascular burden with rates of neurodegeneration in cognitively normal members of the 1946 British birth cohort. Neurology.

[31] Dadar M, Camicioli R, Duchesne S, Collins DL (2020). Alzheimer’s disease neuroimaging I. The temporal relationships between white matter hyperintensities, neurodegeneration, amyloid beta, and cognition. Alzheimers Dement.

[32] VanderWeele TJ (2016). Mediation analysis: a practitioner’s guide. Annu Rev Public Health.

[33] Huang YT, Yang HI (2017). Causal mediation analysis of survival outcome with multiple mediators. Epidemiology.

[34] Nguyen QC, Osypuk TL, Schmidt NM, Glymour MM, Tchetgen Tchetgen EJ (2015). Practical guidance for conducting mediation analysis with multiple mediators using inverse odds ratio weighting. Am J Epidemiol.

[35] Tchetgen Tchetgen EJ (2013). Inverse odds ratio-weighted estimation for causal mediation analysis. Stat Med.

[36] Mathur MB, Ding P, Riddell CA, VanderWeele TJ (2018). Web site and R package for computing E-values. Epidemiology.

[37] VanderWeele TJ, Ding P (2017). Sensitivity analysis in observational research: introducing the E-value. Ann Intern Med.

[38] Smith LH, VanderWeele TJ (2019). Mediational E-values: approximate sensitivity analysis for unmeasured mediator-outcome confounding. Epidemiology.

[39] R Core Team. R: A language and environment for statistical computing. R Foundation for Statistical Computing, Vienna. https://www.R-project.org/.2022

[40] Manschot SM, Brands AM, van der Grond J (2006). Brain magnetic resonance imaging correlates of impaired cognition in patients with type 2 Diabetes. Diabetes.

[41] Moran C, Beare R, Phan T (2017). Neuroimaging and its relevance to understanding pathways linking diabetes and cognitive dysfunction. J Alzheimers Dis.

[42] Espeland MA, Bryan RN, Goveas JS (2013). Influence of type 2 diabetes on brain volumes and changes in brain volumes: results from the women’s health initiative magnetic resonance imaging studies. Diabetes Care.

[43] Moran C, Munch G, Forbes JM (2015). Type 2 diabetes, skin autofluorescence, and brain atrophy. Diabetes.

[44] Janelidze S, Hertze J, Nagga K (2017). Increased blood-brain barrier permeability is associated with dementia and diabetes but not amyloid pathology or APOE genotype. Neurobiol Aging.

[45] Srikanth V, Maczurek A, Phan T (2011). Advanced glycation endproducts and their receptor RAGE in Alzheimer’s disease. Neurobiol Aging.

[46] Abner EL, Nelson PT, Kryscio RJ (2016). Diabetes is associated with cerebrovascular but not Alzheimer’s disease neuropathology. Alzheimers Dement.

