# Supplementary material for: Understanding the relationship between type-2 diabetes, MRI markers of neurodegeneration and small vessel disease, and dementia risk: a mediation analysis
Source: Eur J Epidemiol. 2024 Jan 8;39(4):409–17. doi: 10.1007/s10654-023-01080-7 (PMC11101545; doi:10.1007/s10654-023-01080-7)
Supplement: Supplementary file 1 — Supplementary Material 1 [file 10654_2023_1080_MOESM1_ESM.docx]

| Supplemental Table S1: Baseline characteristics of study participants included in the analysis sample and excluded because of missingness, the 3C study (Bordeaux + Dijon) | | | |
| --- | --- | --- | --- |
|  | Excluded  (n=326) | Included  (n=2,228) | P value* |
| Age at baseline | 73.1 [69.8 ; 76.5] | 72.1 [68.8 ; 75.8] | 0.001 |
| Female | 181 (55.5) | 1340 (60.1) | 0.11 |
| Education level |  |  | 0.11 |
| No diploma or primary school | 100 (30.8) | 686 (30.8) |  |
| Secondary school | 117 (36.0) | 681 (30.6) |  |
| High school | 28 (8.6) | 268 (12.0) |  |
| University | 80 (24.6) | 593 (26.6) |  |
| *Missing data, n* | *1* | *0* |  |
| APOE-ε4 carriers | 75 (25.2) | 479 (21.5) | 0.14 |
| *Missing data, n* | *29* | *0* |  |
| Smoking status |  |  | 0.06 |
| Never smoker | 185 (56.7) | 1368 (61.4) |  |
| Former smoker | 114 (35.0) | 742 (33.3) |  |
| Current smoker | 27 (8.3) | 118 (5.3) |  |
| *Missing data, n* | *0* | *0* |  |
| Drinking status |  |  | 0.06 |
| Non drinker | 70 (22.0) | 373 (16.7) |  |
| Former drinker | 7 (2.2) | 46 (2.1) |  |
| Drinker | 241 (75.8) | 1809 (81.2) |  |
| *Missing data, n* | *8* | *0* |  |
| Baseline MMSE score | 28.0 [26.0 ; 29.0] | 28.0 [27.0 ; 29.0] | <.001 |
| *Missing data, n* | *0* | *5* |  |
| BMI |  |  | 0.01 |
| <20 | 18 (5.5) | 114 (5 .1) |  |
| 20 – 24.9 | 120 (36.9) | 921 (41.3) |  |
| 25 – 29.9 | 125 (38.5) | 915 (41.1) |  |
| >= 30 | 62 (19.1) | 278 (12.5) |  |
| *Missing data, n* | *1* | *0* |  |
| History of CVD | 28 (8.6) | 153 (6.9) | 0.26 |
| *Missing data, n* | *0* | *0* |  |
| History of stroke | 19 (6.0) | 86 (3.9) | 0.08 |
| *Missing data, n* | *34* | *0* |  |
| Diabetes | 37 (13.2) | 181 (8.1) | 0.04 |
| *Missing data, n* | *46* | *0* |  |
| Hypertension | 269 (82.8) | 1691 (75.9) | 0.006 |
| *Missing data, n* | *1* | *0* |  |
| Hypercholesterolemia | 132 (45.0) | 987 (44.3) | 0.81 |
| *Missing data, n* | *33* | *0* |  |
| High depressive symptoms | 54 (16.9) | 237 (10.8) | 0.001 |
| *Missing data, n* | *33* | *0* |  |
| Glycaemia | 4.9 [4.6 ; 5.4] | 4.9 [4.6 ; 5.3] | 0.17 |
| *Missing data, n* | *31* | *0* |  |
| Data presented being N (frequency) or median [IQR]  * p-value : Wilcoxon for quantitative variable or χ2 for qualitative variables  Abbreviations : MMSE : Mini Mental State Examination, APOE : apolipoprotein E, CVD: Cardiovascular disease, BMI: Body Mass Index CVD, BMI | | | |
